# Food banking for improved nutrition of HIV infected orphans and vulnerable children; emerging evidence from quality improvement teams in high food insecure regions of Kiambu, Kenya

**DOI:** 10.11604/pamj.supp.2016.25.2.9663

**Published:** 2016-11-26

**Authors:** Muhamed Akulima, Rudia Ikamati, Margaret Mungai, Muhula Samuel, Meshack Ndirangu, Richard Muga

**Affiliations:** 1 Amref Health Africa, Kenya; 2 Uzima University, Kenya

**Keywords:** Child Status Index, Middle Upper Arm Circumference, food banking, Community, quality improvement, HIV-infected Orphans, vulnerable children

## Abstract

**Introduction:**

Estimated 236,548 People Living with HIV (PLHIV) were in Central-Eastern Kenya in 2013. Kiambu County had 46,656 PLHIV with 42,400 (91%) adults and 4,200(9%) children (1-14yrs). Amref Health Africa in Kenya, supported through USAID-APHIAplus KAMILI project, initiated two food banks to respond to poor nutritional status of the HIV infected children. Quality Improvement Teams were used to facilitate food-banking initiatives. The study aimed at assessing and demonstrating roles of community food-banking in improving nutrition status of HIV-infected children in food insecure regions.

**Methods:**

A pre and post-test study lasting 12 months (Oct 2013 to September 2014) conducted in Kiambu County, Kenya covering 103 HIV infected children. Two assessments were conducted before and after the food banking initiative and results compared. Child Status Index (CSI) and the Middle Upper Arm Circumference (MUAC) tools were used in data collection at households. Paired T-test and Wilcoxon test were applied for analysing MUAC and CSI scores respectively using the SPSS.

**Results:**

There was a significant improvement in the children’s nutrition status from a rating of ‘bad’ in CSI Median (IQR) score 2(2-1) before food banking to a rating of ‘fair’ in CSI Median (IQR) score 3(4-3) after food banking intervention (p=<0.001) while MUAC increased from Mean (SD) of 5.6(2.6) before intervention to 7.2(2.8) after food banking (p=<0.001).

**Conclusion:**

Food banking is a community-based nutritional intervention that can address factors of food access, affordability and availability. Food banking is a sustainable way to contribute to quality nutrition and reduced related deaths among HIV infected children.

## Introduction

In sub-Saharan Africa, children who are HIV-positive are more likely to be stunted, wasted, and underweight [1]. Children with HIV and severe malnutrition tend to have lower nutritional recovery and have higher mortality rates than their HIV-negative counterparts [2]. Those who recover from related illness take longer to achieve nutritional recovery [3]. While there are estimated 137 million children who are under the age of five in sub-Saharan Africa, 12.3million of these are wasted, and an estimated 2.3 million children aged 0-14 have HIV [4]. An estimated 236,5 PLHIV were living in Central- Eastern region of Kenya in 2013. Kiambu County, in the same region had 46,656 PLHIV with 42,400 (91%) being adults and 4,200(9%) children aged 1-14 years [5]. Majority of the children were orphaned due to HIV/AIDS. A total of 140,000 (28%) Orphans and Vulnerable Children were supported by USAID-PEPFAR through APHIAplus KAMILI project in partnership with Amref Health Africa. The Government of Kenya and stakeholders support less than 10% of these children leaving about 60% of them without adequate care and support.

Due to too much focus on the prevention of mother-to-child transmission (PMTCT) to decrease the population of children born with HIV [6], a large percentage of HIV-positive children encounter severe malnutrition as their first AIDS-defining illness [7]. This is due to little emphasis on strategies that address nutrition challenges among the HIV-infected children, mostly beyond the age-five. In the long run, nutrition related deaths have significantly contributed to the higher rate of mortality among the HIV-positive children than their HIV-negative counterparts [2].

Malnutrition is a significant factor affecting human immunodeficiency virus (HIV) care and treatment in resource limited settings and contributes to other infections [8, 9]. Individuals at all stages of HIV disease are at risk of nutritional deficiency and nutritional status is a strong predictor of disease progression, survival and functional status during the course of the disease [10]. Adequate diet is believed to be important for adherence. The poor socioeconomic background of these children often makes the use of commercial formulas for continued nutritional through outpatients impractical [7]. Studies have assessed the impact of food supplements and suggested that nutritional supplementation is acceptable and feasible to improve food intake in malnourished HIV infected patients [11, 12]. Evidence to support the roles and benefits of community food banking in improving the nutritional status of the HIV- infected children in settings with limited food resources is scarce.

Food security is said to exist if at all times, people have a physical and economic access to sufficient, safe and nutritious food that meet their dietary and food preferences, for an active and healthy life [13]. Through Amref Health Africa in Kenya, the consortium partner sub-awarded to implement the child nutrition program, two food banks were established. The food banking was implemented over a period of between 6-12 months. Two assessments were conducted one before and another after the initiation of the food banking and the results compared. The purpose of this study was therefore to assess and demonstrate the roles of local community food banks in improving the nutrition of orphans and vulnerable children who were HIV- infected.

## Methods

### Study design, population and sites

A quantitative design applying a pre and post-test study lasting 12 months (October 2013 to September 2014) conducted in two semi-arid sub-counties, Lari and Ngoliba in Kiambu County, Kenya. The study used a non probability sampling method. The census technique was applied since the entire population was sufficiently smaller (103) and the study was interested in the information of the entire population of the 103 HIV-positive children. All the 103 HIV- infected children aged between 6 months and below 18 years and who were already recruited for HIV care and support services and accessing food banking services in the two sub-counties were included in the study. The sex of the children assessed was 41% male and 59% female. The distribution of the children was 20 children in Lari while Ngoliba had 83 children. Structured questionnaires (forms) and anthropometric measurements were used to collect data through interviews, observation and recording. The project recruited 103 HIV- infected children aged below 18 years during the normal routine household visits by the Community health volunteers. There was a tacit criteria that the child must be eligible for OVC care and support under the USAID PEPFAR guidance, and that the child had not received food assistance from any other program in the previous 12 months prior to the initiation of food banking.

The Child Status Index (CSI) tools [14, 15] and the Middle Upper Arm Circumference (MUAC) [16] tapes were used in data collection at households. The MUAC measurements and the CSI assessments were administered to the individual HIV-positive children in the households concurrently. Caregivers at the households provided supplementary information, besides observations. The main selection criteria for households were that they had HIV-positive children and belonged to either of the two selected sub-counties. The two sub-counties main defining characteristics were erratic rainfall, semi-arid and food scarcity throughout the period of study.

### Ethical review

The study was conducted under a general determination not to be human subject’s research by John Hopkins Bloomberg School of Public Health. It was determined that the study was using the de-identified routine programmatic data to examine HIV, maternal and child, reproductive health, orphan and vulnerable children outcomes in Kenya. Thus, the proposed study did not qualify as human subjects research as defined by DHHS regulations 45 CFR 46.102.

### Data collection

Data collection was done by the program partner staff, the community quality improvement teams (QITs) members and the Community Health volunteers (CHVs) from the Local Implementing partners (LIPs), CHWs, nutrition nurses from the local health centres and supervised by the principal researchers. The data collection was conducted one month before the initiation of the food banks and between 6-12 months after the establishment of the food banks. The program staffs, QITs members and the health workers were trained on the application of the CSI tools, while the nutrition nurses who administered MUAC assessment were oriented on etiquettes of household visits, obtaining verbal consent, administering the questions, scoring and interpretation of the results.

During the training, a pre-test of the tools was done and the data use checked before the actual data collection. The first pre-intervention CSI and MUAC data was collected to record the status of the children before the food banking process. Each HIV-positive child enlisted for the study was assessed using the CSI tool and MUAC tapes. There was a focus on those children who were not benefitting from any other food support program in the region. There were no other restrictions that controlled the beneficiaries from accessing food from the markets, neighbours and relatives.

The CSI data was collected using the CSI form, the information recorded analysed and stored. The CSI data form recorded information on the food access and nutrition wellbeing of the child, while the MUAC assessment aimed at assessing the malnutrition level of the child. The second round of CSI and MUAC data collection, the post-intervention study was conducted after between six months after the initiation of the food bank. The post-intervention data on CSI and MUAC assessment was recorded using the CSI records form while the MUAC tape measurements were recorded in the nutrition nurse diary and transferred into the field note book. Collected data was reviewed daily by the corresponding author and the co-authors before entry and analysis. The MUAC measurements were provided by the local health centre workers who participated in the assessment.

The CSI tools package included the CSI domains guide, CSI records form for scoring, a pictorial version of the domains guide (with translated versions in English and Kiswahili, a Kenyan local language). Using the CSI tools, the wellbeing of the child in food security and nutrition was assessed. The food security factor assessed if the child had sufficient food at all times of the year to grow and have a healthy life. It further assessed the ability of the household to obtain enough food for the child through socially acceptable ways, keeping the child away from begging, stealing, and scavenging for food. The nutrition and growth factor assessed if the child was growing well compared to children of his/her age in the local community. The status of the child was indicated on a ranked scale of 1-2-3-4. The CSI score of 4 (Good) implied that the child’s status or situation was good; there were no concerns and no apparent risk for the child while a status score of 3(Fair) implied the child’s status was generally acceptable, but there were concerns on the part of the caregiver to provide additional resources. A status score of 2(bad) indicated a concern that the child’s status was observably not good and additional services were needed while a score of 1(very bad) implied the child was at serious risk and urgent attention to the child or the situation was needed [14]. The CSI scores for 103 children were tabulated, food security and nutrition domain emerged showing that the HIV- infected children were doing poorly and urgent actions were needed. The observation for supplementary information focused on enquiring on how and when the child ate or got the food.

MUAC assessment recorded the measurement of the middle upper arm circumference to determine the malnutrition level. MUAC is an indicator of wasting and in particular lean body mass [17]. It is a proxy measure of nutrient reserves in muscle and fat. Measurement is not time consuming, and has been documented as an effective predictor of risk of death in children aged 6 to 59 months [18]. MUAC has been successfully used with low-skilled staff given training and supervisory support, and is especially suitable for use in the community. The method is based on a single measurement, as opposed to two measurements (for example weight and height). It does not require heavy material and can be used with a single cut-off for boys and girls [18]. It is increasingly being incorporated into guidelines for the treatment of severe and moderate malnutrition [19]. After the administering, scoring and recording, the CSI scores and MUAC measurements were recorded alongside the child’s name, age and sex analysed and compared. All the completed CSI records forms were collected daily and checked by the program staff to avoid loss or tampering with the information once collected.

### Program of intervention

At the start of the project, QITs were formed at each location level (administrative unit consisting of several villages) to promote and generate innovative community based ideas to address challenges faced in OVC care projects. The QITs comprised of community members, beneficiaries, project staff and the Government of Kenya ministries workers representing the diverse sectors that are sources of support to vulnerable children. Key departments of agriculture, community health, child protection, social workers, volunteers and education sector formed the membership of the QITs. CQITs were trained in quality improvement process, standards, tools and approach. The tools for quality improvement included the CSI, The GoK minimum service standards for quality improvement manual, OVC PEPFAR guidance and Quality improvement Journal. Through the problem analysis and prioritization of problems as part of the quality improvement process, change ideas (appropriate interventions that addressed the quality of food and nutrition) were generated.

Through problem analysis, it was established that the main problems were inadequate rainfall, and storage facilities harvested surplus food. Subsequently, market players bought the surplus at lower prices, hoarded and resold at higher prices during famine. This made the market food unaffordable, inaccessible and destabilized the market prices of food stocks. Two food banks were established, at Ngoliba shopping centre and Lari, both in Kiambu County. Lari food bank, was equipped with solar vegetable drier for drying vegetables during the harvest times. Both food banks were stocked with start-up stocks in cereals and other therapeutic food items. Food banks main roles were to ensure availability, accessibility, stability and affordability at all times throughout the year. The introduction of food banking stabilized the markets for food items and encouraged vulnerable households to produce more since the storage facilities were guaranteed. The food banks applied the ‘banking’ approach and provided services in safe storage of cereals, seeds and vegetables, lending of seeds and food rations. Prices of the food were subsidized while some stock were set aside for free provision to cater for those who could not produce and save in the banks and who were too sick or old to engage in food production and gainful livelihoods.

A total of 96 households with HIV-positive children, among other vulnerable households received ‘food banking’ services. All the 103 HIV-positive children and their households were linked to the food banks and sensitized on the importance of food banking and the linkages. The beneficiary’s main roles were to deposit, withdraw, borrow and repay seeds and cereals with ‘interest’ in the form of seeds and cereals. The stock depositors were allowed to ‘withdraw’ during famine. A 10% service charge was levied for deposited cereals, seeds or for drying of vegetables to sustain the operations of the food bank. The food banks were linked to local commercial millers for stock management. The children were also linked to supplemental and therapeutic nutrition support to boost overall health and energy, provide immune system support, increase efficacy of other medical treatment, improve general quality of life and support the healing process [20].

### Statistical analysis

The HIV- infected children’s demographic information was compiled showing their ages and sex. The CSI and the MUAC scores for each child distributed by site and implementation partner were compiled. The demographic tables showed the distribution of the 103 HIV-positive children in the CSI scores of 1to 4 and as well as the MUAC measurement before and after the interventions in food banking. The MUAC measurements were interpreted on a scale ranked from severe Acute Malnutrition (SAM), Moderate Acute Malnutrition (MAM), Mild-at-Risk of malnutrition and normal [18]. In the assessment, MUAC <11.5cm for the ages 6-59 months was ranked with colour Red as Severe Acute Malnutrition (SAM) [18]. Similarly, MUAC<12.9cm for ages 5-9years, MUAC <16.0cm for ages 10-14, and MUAC <22.0cm for ages 15-18 were rated as SAM [21, 22]. Children ranked as severely malnourished had a clinical implication that a real threat of death or profound long term nutrition related complications were eminent. Such children would require that they be hospitalised and provided with therapeutic milk, resuscitation and antibiotics [18]. In extreme cases they may require blood transfusion. In addition MUAC measurements >13.5cm for 6-59months, 14.5 for 5-9years,>18.5cm for ages 10-14years and MUAC>24.5cm for those above 15 years implied normal nutrition status [23].

The paired T-test was applied for analysing MUAC [18] and while the Wilcoxon rank test was applied in the analysis of CSI scores. The analysed CSI data was presented using medians with inter quartile ranges while the MUAC data was analysed and presented using the mean with standard deviations. The study compared the changes in the individual child CSI and MUAC assessment scores in the six-twelve months of intervention in food banking using the Wilcoxon rank test and the paired T-test respectively. The decrease in the proportion of those whose scores were very bad(CSI=1) and bad (CSI=2) and the increase in the proportion of those whose status had changed to fair (CSI=3) and good (CSI=4) were the key factors in the analysis of improvement in the nutrition status using the CSI. The change in the MUAC median and CSI mean with a p value p=<0.001 was considered significance in our analysis.

**Ethical approval:** for this type of study formal consent was not required; this article does not contain any studies with human participants or animals performed by any of the authors. Informed consent: informed consent was obtained from all individual participants included in the study.

## Results

In total 103 HIV-infected children were assessed with CSI and MUAC tapes. All the 103 HIV- infected children completed the 12 months study for both CSI and MUAC and were present for the whole period up to 12 months. Both CSI and MUAC assessments were administered to all the 103 HIV- infected children. As shown in Table 1, the HIV-positive children had varied demographic characteristics of age, sex, and CSI and MUAC rankings. There were more females at 59.2% (n=103) than males 40.8 % (n=103).

**Table 1:** Distribution of the HIV infected children by sex & by percentage (n=103)

Sex and age	Distribution of HIV infected children by number (percentage %)
**Male**	42 (40.8)
**Female**	61(59.2)
**Distribution by age& by percentage ( n=103)**	
**≤ years (0-60months)**	19 (18.4)
**5-9 years**	45 (43.7)
**10-14 years**	31 (30.1)
**15-19years**	8 (7.8)

In 201/3/14 HIV infected children were classified by age and gender. Source; Ngoliba VWB& Cheer Up CBO data base

### Improvements in CSI and MUAC scores after food banking

At between 6-12months on nutritional care through food banking and linkages, children demonstrated changes in their MUAC and CSI status, often as improvements in their nutrition status. Results from the CSI analysis showed there was a significant improvement in the children’s nutrition status from a rating of ‘bad’ in CSI Median (Interquartile Range IQR) score 2(2-1) before food banking to a rating of ‘fair’ in CSI Median (IQR) score(4-3) after food banking intervention (p=<0.001). As shown in Table 2, in food security, those rated as very bad (CSI=1) reduced from 41.7% to 0% and bad(CSI=2) reduced from 47.6% to 0%, fair (CSI=3) increased from 9.7% to 53.4% and those rated well(CSI=4) increased from 9.7% to 46.6%.

**Table 2:** Food security CSI scores and changes between pre and post food banking intervention by percentage (n=103)

CSI ratings	HIV infected children by number (percentage %) before Food banking interventions	HIV infected children by number (percentage %) after food banking interventions	Percentage change
Very bad (CSI=1)	43(41.7)	0(0)	-41.7
Bad (CSI=2)	49(47.6)	0(0)	-47.6
Fair (CSI=3)	10(9.7)	55(53.4)	43.7
Good (CSI=4)	1(1.0)	48(46.6)	45.6
Total	103(100)	103(100)	

In 2013/14 Food security of the HIV infected children was assessed using CSI index. CSI Scores were collected before and after food banking initiatives and compared to measure the changes and improvement. Source; Ngoliba VWB& Cheer Up CBO periodic CSI data

Table 3 presents nutrition and growth CSI scores and changes between pre and post food banking intervention. In nutrition and growth factor, those rated as very bad (CSI=1) reduced from 47.6% to 0% and bad(CSI=2) reduced from 52.4% to 0%, fair (CSI=3) increased from 0% to 51.5% and those rated as good(CSI=4) increased from 0% to 48.5%.

**Table 3:** Nutrition and growth CSI scores and changes between pre and post food banking intervention (n=103)

CSI ratings	HIV infected children by number (percentage %) before Food banking interventions	HIV infected children by number (percentage %) after food banking interventions	Percentage change
Very Bad (CSI=1)	49(47.6)	0(0)	-47.6
Bad (CSI=2)	54(52.4)	0(0)	-52.4
Fair (CSI=3)	0(0)	53(51.5)	51.5
Good (CSI=4)	0(0)	50(48.5)	48.5
Total	103(100)	103(100)	

In 2013/14 Nutrition and Growth of the HIV infected children was assessed using CSI index. CSI Scores were collected before and after food banking initiatives and compared to measure the changes and improvement. Source; Ngoliba VWB& Cheer Up CBO periodic CSI data

The MUAC increased from a Mean (SD) of 5.6(2.6) before intervention to Mean (SD) of 7.2(2.8) after food banking intervention (p=<0.001). Table 4 presents MUAC scores for pre and post food banking interventions The MUAC assessment results showed that those rated as SAM reduced from 25.2% to 3.9% while those rated as MAM reduced from 18.4% to 9.7%. Those rated as Mild-at-risk increased from 15.5% to 24.3% and those rated normal increased from 40.8% to 62.1 %.(n=103). Table 5 presents improvements in MUAC scores after food banking interventions. A significant proportion of malnourished children improved from more severe towards less severe malnutrition categories after food banking. A total of 6.8 %( n=103) of the children improved from SAM to MAM, 10.7% from SAM to moderate at risk while 3.9% improved from SAM to normal. SAM category had the highest combined proportion of children (21.4%) who moved from more severe malnutrition towards less severe malnutrition categories, followed by MAM(15.5%) and Mild-at-risk (12.6%). In summary, a combined proportion of 52.4% of children improved in their MUAC rankings while 47.6% maintained their rankings after food banking, with 40.8% maintaining their normal status MUAC ranking.

**Table 4:** MUAC scores for HIV infected children at pre and post food banking interventions (n=103)

MUAC Ranks	HIV infected children by number (percentage %) before Food banking interventions	HIV infected children by number (percentage %) after food banking interventions	Percentage change
Severely Acute Malnutrition(SAM)	26(25.2)	4(3.9)	-21.3
Moderate Acute Malnutrition (MAM)	19(18.4)	10(9.7)	-8.7
Mild-At risk Malnutrition	16(15.6)	25(24.3)	8.7
Normal	42(40.8)	64(62.1)	21.3
Total	103(100)	103(100)	

In 2013/14 Nutrition of the HIV infected children was assessed using MUAC tapes. MUAC measurements were collected before and after food banking initiatives and compared to measure the changes and improvement. Source; Ngoliba VWB& Cheer Up CBO periodic MUAC data

**Table 5:** Improvements in MUAC after food banking interventions (n=103)

MUAC categories (Movement across ranks)	HIV infected children by number (percentage %)
Severely Acute Malnutrition to severely Acute Malnutrition	4(3.9)
Severely Acute Malnutrition to moderate	7(6.8)
severely Acute Malnutrition to Mild-at Risk	11(10.7)
severely Acute Malnutrition to normal	4(3.9)
Moderate to moderate	3(2.9)
Moderate to normal	10(9.7)
Moderate to Mild-at risk	6(5.8)
Mild-at risk to normal	13(12.6)
Mild-at risk to Mild at risk	3(2.9)
Normal to normal	42(40.8)
Total	**103**(100)

In 2013/14 Nutrition of the HIV infected children was assessed using MUAC tapes. MUAC measurements were paired progressively to determine the proportion of those children whose MUAC improved from one category to the other. Source; Ngoliba VWB& Cheer Up CBO periodic MUAC data

## Discussion

In this study, we have assessed the changes in the nutritional status of HIV infected children in the context of food banking using some of the modern tools applied in measuring malnutrition as response to HIV/AIDS pandemic. The study observed a higher combined number of malnourished HIV-infected children at 59.2% (SAM, MAM and Mild-at- risk) against 40.8% normal at the start and prior to the initiation of the food banks. At the post –intervention, the proportion of children categorized as having normal nutrition status (62.1%) was greater than those categorized as malnourished when the SAM, MAM and Mild-at-Risk (37.9) categories were combined. The pre-intervention situation was consistent with the study reports that associated the significantly higher risk of malnutrition among children infected than among non-infected children [24].

The study observed improvement in the nutritional status of the HIV- infected children using two parameters; the CSI and the MUAC measurements. By 6-12 months after the initiation of food banks, the study demonstrated children nutritional status improvement through movement from CSI rankings of very bad (CSI=1)and bad (CSI=2) to CSI rankings of fair(CS1=3) and good(CSI=4). There was no death related to malnutrition reported during the study period. The MUAC measurements showed increasing number of children moving from more severe malnutrition categories to lesser malnutrition categories of moderate and normal malnutrition at the end of the study period. Greatest improvement in the nutritional status was observed in children with MUAC rankings of SAM and normal. Those who had ranked as SAM at the start of the study period reduced by 21.3% from 25.2% to 3.9% while those who were ranked as normal increased by 21.3% from 40.8% to 62.1%. This suggest that nutritional support through food banking initiatives led to the improved nutritional status and growth for HIV-infected OVC by ensuring access to affordable, high nutritious food on time. These findings resonates the study reports that food assistance, provided to households of ART-naive PLHIV, significantly improved BMI, MUAC, and household food security, compared to others not provided with food assistance [25].

In this study, we observed that community-based management for malnutrition is now considered the standard of care for children with uncomplicated malnutrition, which accounts for more than 90% of cases of severe malnutrition, for children who demonstrate an appropriate appetite and have reliable caregivers [3]. The improvement in nutrition status for HIV-infected children among those served through food banks is a sufficient indication of the need to prioritize and promote food banking in food insecure regions. Governments and food security stakeholders will need to strengthen and integrate food banking in their interventions, with emphasis on community participation and linkages to health systems. A community participatory approach in food banking is recommended due to its potential to yield a range of advantages. The participatory approach will ensure wider community acceptance, consumption and ultimate success of food banking services. Further, since food banks success is hinged on the continuous food stocks deposits, withdrawals, borrowing and repayments, greater community participation in food banking initiatives will result into sustainability, continuity and viability, while meeting the nutrition needs of the general populations and for the People Living with HIV.

The findings from this study supports the study reports by Nalwoga [24] who observed that the response to malnutrition (undernutrition) in children in Africa involves action on diverse fronts, including delivery of community-wide HIV and nutritional interventions as well as addressing the many interacting factors that contribute to childhood undernutrition. Food banking directly fits this description as a nutritional intervention that addresses factors of food access, affordability and availability.

The study further observed that 3.9% of SAM, 2.9% of MAM categories did not have any significant changes in their rankings. A possible explanation for this was that the children required a different nutrition intervention to be initiated prior, alongside or alternative to food banking. There was no any change for 40.8% who maintained their normal status. Possibly, food banking helped maintain the normal status or the children had already overcome nutrition challenges and no longer needed nutritional support. Again, it is possible that results would be different if the study was conducted in settings without food banking and other food sources that sustain proper nutrition

In this study, there were no significant differences observed in the MUAC measurements for females and males. This was because the MUAC measurement does not require heavy material and can be used with a single cut-off for boys and girls [19]. In case of differences, then a possible explanation could be that the age cohorts were not well demarcated and comparisons were therefore made between boys and girls with wider age differences.

### Strengths and limitations

The study’s main strength was the use of MUAC and the CSI to measure the nutrition levels of the HIV- infected children in a community setting. MUAC has been successfully used with low-skilled staff given training and supervisory support, and is especially suitable for use in the community. The method is based on a single measurement, as opposed to two measurements for example weight and height [18]. The CSI has been successfully applied and validated as a reliable measure for nutritional status of children [14, 15].

The first study limitation was the relative smaller sample (census), shorter period between the start and end of study may have a limiting interpretation and application of the findings. A study period or a food banking period of over one year will allow adequate follow-up for concrete outcomes assessments. Second, the exclusion of the children not infected with HIV who may present similar malnutrition symptoms despite their HIV-negative status limits the study. HIV-infected children assessed alone for nutritional improvement through food banking may present confounded symptoms that may be due to intolerance to clinical food supplementation and HIV Anti-Retroviral Treatment and this will make it difficult to draw conclusions if it’s the effect of HIV treatment or nutrition improvement. Very few studies have investigated food banking initiatives and outcomes measured through CSI and MUAC. Despite the study limitations, this study contributes evidence that greatly contributes to the enquiries and practice on the role of community food banking in improving the nutritional status of HIV-infected children, more so through a community participatory approach.

## Conclusion

This study demonstrates that food banking as a community-based nutritional intervention can address factors of food access, affordability and availability for HIV-infected children and their households. Food banking has the potential to be imperative part of standard of quality care for HIV infected children in food insecure regions. Initiating food banking through community participation contributes to sustainable and better access to quality nutrition for HIV-infected children and can reduce malnutrition related death. A further study on food banking incorporating HIV un-infected children is recommended to yield further evidence, validate this study and inform policy formulation.

**What is known about this topic**

- There is significantly higher risk of malnutrition among HIV infected children than among non-infected children;
- Food assistance, provided to households of ART-naive PLHIV, significantly improves BMI, MUAC, and household food security;
- Community-based management for malnutrition is now considered the standard of care for children with uncomplicated malnutrition.

**What this study adds**

- New insights and evidence on how food banking can directly address factors of food access, affordability and availability;
- Descriptive set of Knowledge, process, structure and management of food banking initiatives; No any studies done on this;
- A new socio-scientific descriptive methodological perspective on the use of dual parametric measurement and assessment of malnutrition using MUAC and CSI; No evidence of any other study having applied both at the same time. A new replicable, scalable and sustainable food banking approach for nutritionists, care and service providers working for children
